# Characterization of Senecavirus A Isolates Collected From the Environment of U.S. Sow Slaughter Plants

**DOI:** 10.3389/fvets.2022.923878

**Published:** 2022-06-22

**Authors:** Kyle S. Hoffman, Nicki L. Humphrey, John A. Korslund, Tavis K. Anderson, Kay. S. Faaberg, Kelly M. Lager, Alexandra C. Buckley

**Affiliations:** ^1^Virus and Prion Research Unit, National Animal Disease Center, U.S. Department of Agriculture, Agricultural Research Service, Ames, IA, United States; ^2^Veterinary Services, U.S. Department of Agriculture, Animal Plant Health Inspection Service, Fort Collins, CO, United States; ^3^Veterinary Services, U.S. Department of Agriculture, Animal Plant Health Inspection Service, Riverdale, MD, United States

**Keywords:** Senecavirus A, genetic diversity, slaughter plants, bioassay, SVA, swine

## Abstract

Vesicular disease caused by Senecavirus A (SVA) is clinically indistinguishable from foot-and-mouth disease (FMD) and other vesicular diseases of swine. When a vesicle is observed in FMD-free countries, a costly and time-consuming foreign animal disease investigation (FADI) is performed to rule out FMD. Recently, there has been an increase in the number of FADIs and SVA positive samples at slaughter plants in the U.S. The objectives of this investigation were to: (1) describe the environmental burden of SVA in sow slaughter plants; (2) determine whether there was a correlation between PCR diagnostics, virus isolation (VI), and swine bioassay results; and (3) phylogenetically characterize the genetic diversity of contemporary SVA isolates. Environmental swabs were collected from three sow slaughter plants (Plants 1-3) and one market-weight slaughter plant (Plant 4) between June to December 2020. Of the 426 samples taken from Plants 1-3, 304 samples were PCR positive and 107 were VI positive. There was no detection of SVA by PCR or VI at Plant 4. SVA positive samples were most frequently found in the summer (78.3% June-September, vs. 59.4% October-December), with a peak at 85% in August. Eighteen PCR positive environmental samples with a range of C_t_ values were selected for a swine bioassay: a single sample infected piglets (*n* = 2). A random subset of the PCR positive samples was sequenced; and phylogenetic analysis demonstrated co-circulation and divergence of two genetically distinct groups of SVA. These data demonstrate that SVA was frequently found in the environment of sow slaughter plants, but environmental persistence and diagnostic detection was not indicative of whether a sampled was infectious to swine. Consequently, a more detailed understanding of the epidemiology of SVA and its environmental persistence in the marketing chain is necessary to reduce the number of FADIs and aide in the development of control measures to reduce the spread of SVA.

## Introduction

Senecavirus A (SVA) is a non-enveloped, single-stranded, positive-sense RNA virus in the family *Picornaviridae*. It is the only species in the genus *Senecavirus* and is most closely related to viruses in the genus *Cardiovirus* (1). The SVA genome is approximately 7.2 kb long and encodes a single large polyprotein which is cleaved by the viral cysteine protease 3C (2). The polyprotein is composed of four structural proteins VP1, VP2, VP3 and VP4 and eight non-structural proteins L, 2A, 2B, 2C, 3A, 3B, 3C and 3D (1). Senecavirus A was discovered as a cell culture contaminate in 2002 and named Seneca Valley virus (SVV-001) (3). Between 1988 and 2005 picorna-like viruses were isolated from pigs displaying various clinical symptoms (4). Samples were partially sequenced and were similar to SVV-001, revealing that SVA has been present in swine populations in the United States for at least thirty years (4). Subsequently, SVA has been identified and described in swine from Brazil, Canada, China, Thailand, Vietnam and Colombia (5–10).

In 2015, experimental studies determined SVA was a causative agent for vesicular disease in pigs, which is characterized by blister-like lesions found on the snout and coronary band (11–13). Vesicular lesions resolve in 1–2 weeks, while viral nucleic acid can continue to be detected in oral/nasal and rectal swabs for several weeks after infection (13, 14). However, virus isolation from swab samples has typically only been reported during the first week after experimental infection. Recently, SVA was isolated from the tonsil of animals after a stressor event (i.e., transportation or parturition) 60 days after experimental infection, providing experimental evidence that SVA may establish a persistent infection in the tonsil of infected swine (15).

Both experimental and field studies have demonstrated transmission of SVA from pig-to-pig through direct contact (15, 16). In addition, viable SVA has been recovered from environmental samples and mouse feces, and viral nucleic acids have been recovered from house flies, potentially providing other routes for transmission of SVA to susceptible hosts (17). SVA has been detected in both swine arriving to slaughter plants as well as assembly yards (18, 19). The presence of SVA in areas where swine from multiple origins congregate may have broad impacts, as contaminated transport vehicles could be a source of viral transmission back to swine farms (20–22). Additional evidence suggests that transportation stress may increase clinical disease severity and this may be relevant in sows shipped to slaughter plants (15).

SVA and foot-and-mouth disease (FMD) cause clinically identical vesicular disease, and the presence of vesicular lesions on swine requires a foreign animal disease investigation (FADI) in FMD free countries (23). With an increase in the number of SVA cases in the U.S. since 2015, particularly centered at slaughter plants, there has been a corresponding increase in FADIs. These investigations impart a substantial burden on local and federal resources and can disrupt market supply chains, thus further adding to the cost (24). Therefore, a better understanding of SVA ecology at slaughter plants could inform measures to reduce the number of FADIs due to SVA. The objectives for this study were to understand the environmental burden of SVA in sow slaughter plant lairages; to determine whether there was a correlation between PCR detection, viral isolation (VI), and swine bioassay results; and to genetically characterize 2020 SVA sequences from environmental samples to prior SVA isolates circulating in the United States.

## Materials and Methods

### Samples

Environmental samples were collected between June and December 2020 from three U.S. sow slaughter plants in the Midwest that process between 500 and 2,800 head/day. Plant 4 was a market-weight slaughter plant also in the Midwest that processes ~12,000 head/day. Plants 1-3 had previously experienced a high incidence of FADIs and Plant 4 had no reports of FADIs. Cotton-tipped swabs used for environmental collection were pre-moistened with tris-buffered tryptose broth (TBTB, 1.21 g/L Tris-base, 26 g/L Tryptose broth). Individual swabs were gently rubbed on surfaces exposed to animals that included flooring, waterers, gating, side panels of trailers and the coronary bands of pigs without vesicular lesions and then placed into 2 mL of TBTB media. Samples were shipped to the National Animal Disease Center (NADC) in Ames, IA. Tubes containing environmental swabs were clarified to pellet residual debris and supernatant was filtered through a 0.45 μm filter, aliquoted, and frozen at−80°C for future testing.

### SVA Nucleic Acid Extraction and Quantification

Environmental swab samples and samples from the swine bioassay were tested for SVA nucleic acids by reverse transcription-quantitative PCR (RT-qPCR) as previously described (14). Briefly, RNA was extracted from samples using the MagMAX Pathogen RNA/DNA kit (Applied Biosystems, Waltham, MA) following the manufacturers' recommendations. Subsequently, 5 μL was added to 20 μL of the Path-ID Multiplex One-Step RT-PCR reaction master mix for rectal swabs and environmental swabs or 20 μL AgPath-ID One-Step RT-PCR kit (Applied Biosystems) for oral swabs and sera. The primers and probe were designed to target a conserved region containing nucleotides 602-710 of the SVA genome. The forward primer sequence was 5′-TGCCTTGGATACTGCCTGATAG-3′, the reverse primer sequence was 5′-GGTGCCAGAGGCTGTATCG-3′, and the probe sequence was 5′-CGACGGCCTAGTCG GTCGGTT-3′. RNA copies were determined using a dilution series with a plasmid containing the target region and cycle threshold (C_t_) values >35 were considered negative.

### Cells and Virus Isolation

Swine testicular (ST) cells (National Veterinary Services Laboratory, Ames, IA) were grown in minimum essential media (MEM, Gibco, Waltham, MA) supplemented with 10% fetal bovine serum (FBS, Atlanta Bio, Flowerly Way, GA), 1% L-glutamine (Life Technologies, Carlsbad, CA) and 50 mg/L gentamicin at 37°C and 5% CO_2_. Monolayers of ST cells were grown until confluent in 24 well plates, MEM was removed and replaced with 1 mL serum-free MEM. Cells were inoculated with 100 μL of filtered environmental samples and allowed to incubate at 37°C and 5% CO_2_ for 1 h, after which the inoculum was removed and replaced with 1 mL MEM. Negative control wells were present on each plate. Plates were microscopically observed daily for 4 days for cytopathic effect (CPE). Two additional blind passages were performed on each environmental sample.

### Swine Bioassay Design in 3–5-Day-Old Pigs

All animal research was performed in accordance with an Animal Care and Use Protocol (ACUP ARS-2018-750) approved by the NADC Animal Care and Use Committee. At the end of the study, all animals were humanely euthanized with an intravenous administration of a barbiturate (Fatal Plus, Vortech Pharmaceuticals, Dearborn, MI) following the label dose (1 mL/4.45 kg).

Eighteen SVA PCR positive environmental samples were selected for swine bioassay (Table 1). Samples were representative of the various slaughter plants, sample types, and dates of collection. Two samples, one VI positive and one VI negative, were selected for each C_t_ value between 23 and 31. The exception was the C_t_ value of 23 which did not have any VI negative samples. Environmental samples were diluted 1:5 with serum free MEM to generate material for the swine bioassay, PCR, and VI.

**Table 1:** RT-qPCR, virus isolation (VI), and swine bioassay results of selected SVA samples from Plants 1-3.

**Plant #**	**Date**	**Sample type**	**RT-qPCR C_**t**_ value**	**Genomic copies/mL**	**VI Result**	**Bioassay**
Plant 3	09/08/20	Waterer	24.18	1.14E+06	+	2/2
Plant 1	10/29/20	Flooring	24.29	1.06E+06	+	0/2
Plant 3	09/14/20	Flooring	25.99	3.49E+05	+	0/2
Plant 3	07/27/20	Pig	26.72	1.84E+05	+	0/2
Plant 1	06/24/20	Flooring	26.85	1.98E+05	+	0/2
Plant 1	09/09/20	Flooring	26.95	1.85E+05	+	0/2
Plant 2	08/28/20	Pig	27.23	1.54E+05	−	0/2
Plant 2	07/22/20	Gating	27.56	1.02E+05	+	0/2
Plant 3	07/27/20	Waterer	28.03	7.26E+04	+	0/2
Plant 1	07/21/20	Gating	29.75	2.16E+04	−	0/2
Plant 1	09/09/20	Trailer	30.83	4.00E+04	−	0/2
Plant 3	08/31/20	Flooring	31.07	8.62E+03	+	0/2
Plant 1	06/30/20	Flooring	31.13	8.11E+03	−	0/2
Plant 1	06/24/20	Flooring	31.20	7.74E+03	−	0/2
Plant 2	07/22/20	Waterer	32.01	4.39E+03	−	0/2
Plant 3	08/10/20	Gating	32.08	4.33E+03	+	0/2
Plant 3	08/10/20	Waterer	32.50	3.13E+03	+	0/2
Plant 2	07/14/20	Flooring	33.14	2.09E+03	−	0/2

Piglets were weaned between 3 and 5 days-of-age and placed into individual isolator cages with two cages per ABSL-2 animal space. After 24 h of acclimation, piglets were bled, oral and rectal swabbed, and inoculated (*n* = 2/sample) with 2 mL orally on 0 days post inoculation (dpi). All animals were observed daily for clinical signs including lameness, lethargy, inappetence and diarrhea. Blood, oral swabs, and rectal swabs were collected on 6 dpi when piglets were removed from isolation cages and placed on raised decks in the same ABSL-2 animal space, as well as on 10 and 14 dpi (12, 13). Blood was collected in serum separator tubes (BD Vacutainer, Franklin Lakes, NJ) and centrifuged to separate the serum for storage. Oral and rectal swabs were collected using a sterile polyester tipped applicator (Puritan Medical Products, Guilford, ME) immersed in 3 mL of MEM. All samples were frozen at −80°C prior to testing.

### Virus Neutralization Assay

Serum samples from 0, 4, 6 and 10 dpi were heat inactivated at 56°C for 30 min. Samples were run in quadruplicate, diluted four-fold from 1:4 to 1:4096 in MEM. Cross-neutralizing antibodies have been demonstrated with different geographic and temporal isolates of SVA (25), as such a reference isolate of SVA (SVA/KS/2018) was selected for the VN assay. An equal volume of serum and SVA isolate (~200 TCID_50_) were mixed and incubated for 1 h at 37°C. The virus-serum mixture was transferred to 96-well plates of confluent ST cells with growth media replaced by MEM supplemented with 2% FBS. Plates were microscopically evaluated daily for 4 days. The titer was recorded as the highest dilution of serum where viral infectivity was completely neutralized in 50% of the inoculated wells. A back titration of the SVA isolate was performed for each run. Virus neutralization titers of ≤ 1:16 were considered negative.

### Sequencing

Six environmental samples were selected for whole genome sequencing. Two samples with the lowest C_t_ values were selected from Plants 1, 2 and 3 and 100 μL of each sample was used to inoculate ST cell monolayers in 25 cm^2^ flasks for 1.5 h. Flasks were decanted, replaced with serum-free MEM, and incubated for 24–48 h. Flasks were frozen at−80°C and three freeze/thaw cycles were performed prior to clarification at 3,000 XG for 10 min. Clarified cell culture supernatant was submitted to the Iowa State University Veterinary Diagnostic Laboratory for next generation sequencing using the Illumina platform. Sample library preparation and sequencing were performed following previously described methods (26). The accession numbers for the SVA genome sequences are as follows: NADC1 (MZ733980), NADC2 (MZ733979), NADC3 (MZ733978), NADC4 (MZ733977), NADC5 (MZ73396) and NADC6 (MZ733975).

### Sequence Analysis, Alignment and Phylogenetic Analysis

Illumina sequence reads from environmental samples were imported into Geneious Prime^®^ 2021.2.2 and were paired using default conditions. Paired-end reads were mapped to a reference SVA sequence (MT360257.1). Publicly available full-length SVA genome sequences (>7,100 bp) with location and date of collection were downloaded from NCBI GenBank on August 14 2021 (27). The complete genome sequences of SVA isolated in this study along with the full-length SVA genomes available on GenBank were aligned using MAFFT with default settings (28). A maximum-likelihood phylogenetic tree for the SVA genomes was inferred using IQ-TREE following automatic model selection on the IQ-TREE web server (29): statistical support was assessed with 1,000 ultrafast bootstrap iterations.

To estimate the evolutionary dynamics of the SVA genome sequences collected in the United States after 2002, we implemented a time-scaled Bayesian approach. All SVA genome sequences collected in the U.S. were downloaded from NCBI GenBank on October 15, 2021 and aligned using default settings in MAFFT. These data were screened for evidence of recombination in RDP5 with the application of the following algorithms RDP, MaxChi, 3seq, GENECOV, Bootscan, SiScan, and Chimera (30). Genomes were considered to have a recombination signal when more than four of the above methods had statistically significant support for recombination, when the identified breakpoints and breakpoint regions were longer than 100 nucleotides and when the parental and recombinant strains exhibited spatial and temporal concordance [e.g., (31)]. Recombinant sequences were removed from subsequent Bayesian analyses. A maximum likelihood phylogenetic tree was inferred with FastTree (32) from the resultant dataset (*n* = 110), and screened in TempEst (33) to assess whether there were any sequences with incongruent dates and divergence resulting in a final dataset of 89 sequences. A time-scaled Bayesian phylogenetic tree was inferred in BEAST v1.10.4 (34) employing a strict molecular clock, a coalescent-based constant size demographic model (35) and a HKY85 + Γ substitution model. The Markov chain Monte Carlo (MCMC) was run for 100 million steps with sampling every 10,000 generations. Two independent analyses were performed, and convergence was assessed in Tracer v1.7.2. Evolutionary history was summarized and visualized using an annotated maximum clade credibility tree using TreeAnnotator v1.8.4 and FigTree v1.4.4.

## Results

### RT-qPCR and VI Results of Environmental Samples

**Figure 1:**
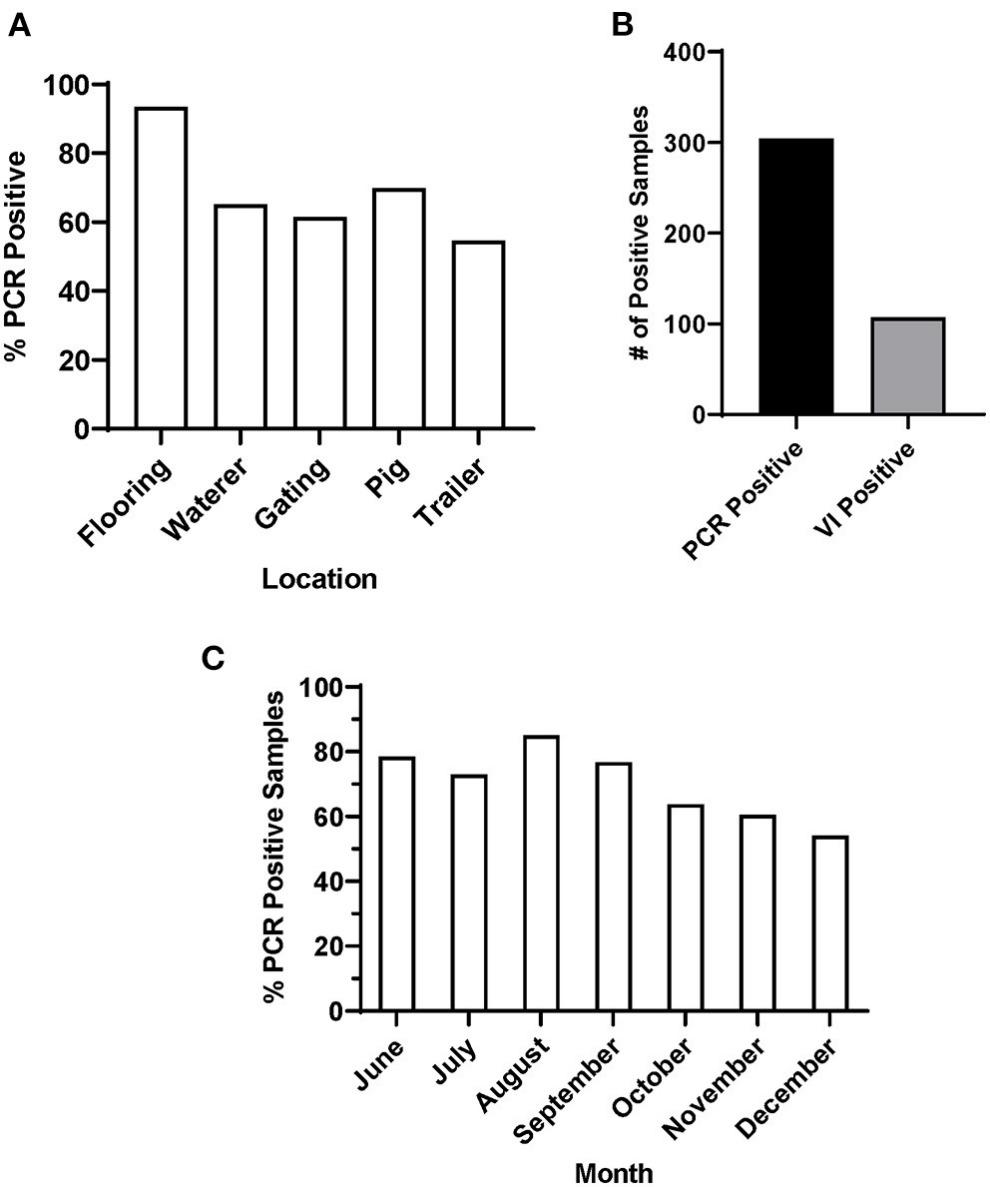
PCR and VI results of environmental samples from Plants 1-3. **(A)** Percentage of SVA positive samples by sample location, **(B)** total number of SVA PCR positive and VI positive samples and **(C)** percentage of SVA positive samples by month.

In this study, environmental swab samples were collected from three sow slaughter plants, with high incidence of FADIs (Plants 1-3) and a fourth market-weight slaughter plant with no reported FADIs (Plant 4). All samples (*n* = 69) tested from Plant 4 were negative for SVA by RT-qPCR. From Plants 1-3, 426 swab samples were taken from the environment including flooring (*n* = 92), waterers (*n* = 92), gating (*n* = 91), trailers (*n* = 22) and from the coronary bands of pigs (*n* = 129) that did not have vesicular lesions. Data for Plants 1–3 were combined for subsequent analyses. For each sample type, <50% of the samples collected across the sampling period were RT-qPCR positive for SVA. Samples collected from the flooring of the lairage had the highest number of percent positives (93.5% positive), while samples collected from gating (61.5% positive) and trailers (54.6% positive) were the two lowest percent positive sample types (Figure 1A).

Of the 71.4% of environmental samples that were SVA RT-qPCR positive, 35.2% were also VI positive (Figure 1B). Samples with a lower C_t_ value were more likely to be VI positive. 67.6% of all samples with a C_t_ value below 30 were VI positive, while only 18.8% of samples with a C_t_ value between 30 and 35 were VI positive. These data were subsequently separated by season to determine whether there was any pattern in SVA percent positive across the sampling period. When all sample data were combined by month, the percentage of positive samples for Plants 1-3 SVA was higher throughout the summer, with a percent positive peak in August (85%), that then declined through the fall and early winter (Figure 1C).

### Swine Bioassay

A swine bioassay was performed to determine whether there was a correlation between PCR C_t_ values, virus isolation and SVA infectivity in swine. The inoculum was tested by PCR and VI the same time as animals were inoculated. SVA RT-qPCR C_t_ values for the inoculums ranged from 24 to 33 and 11/18 were VI positive (Table 1). Lower C_t_ values were more likely to be VI positive, but some inoculums with higher C_t_ values (~32) were also VI positive.

Pigs used in the swine bioassay tested negative for SVA nucleic acids and neutralizing antibodies prior to inoculation. Only one set of piglets were infected with SVA and had positive swabs and serum at 6 dpi when removed from isolation cages. These two pigs also developed neutralizing antibody titers of 256 by 14 dpi. The inoculum material had the lowest C_t_ value (24.18), tested positive for virus isolation, and came from an environmental sample collected from a waterer in the lairage (Table 1). Remaining pigs were RT-qPCR negative for SVA in all samples collected and there was no evidence of a neutralizing antibody response in 14 dpi sera.

### SVA Whole Genome Sequence Analysis

Six environmental samples (*n* = 2/plant) with the lowest C_t_ values were selected for whole genome sequencing. A complete SVA polyprotein and the accompanying 5′ and 3′ UTR nucleotides were determined for each of the six samples with no insertions or deletions. The pairwise identity between all six sequences was 98.2%. Percent identity between the nucleotide and amino acid sequences of the six SVA isolates ranged from 96.5 to 99.9% and 98.9 to 99.9%, respectively. NADC1 was most dissimilar from the other five isolates sequenced with approximately 250 nucleotide differences across the genome (Table 2). When excluding NADC1, the remaining isolates had 99.0 and 99.7% identity at the nucleotide and amino acid level, respectively.

**Table 2:** Complete genome sequence distance matrix and heat map for the six sequenced SVA isolates.

	**NADC6**	**NADC5**	**NADC4**	**NADC3**	**NADC2**	**NADC1**
NADC6		108	58	112	113	248
NADC5	98.5		88	20	25	249
NADC4	99.2	98.8		89	93	246
NADC3	98.5	99.7	98.8		12	250
NADC2	98.5	99.7	98.7	99.9		253
NADC1	96.6	96.6	96.6	96.6	96.5	

*The black bars indicate 100% identity between homologous isolates, above these black bars indicates number of nucleotide differences between the isolates and below the black bars indicates percent nucleotide identity between the isolates*.

There was a significant increase in the detection of SVA cases in the U.S. during 2015. To identify if any conserved non-synonymous amino acid changes had occurred since 2015, the 2020 SVA isolate genomes were compared to 2015 U.S. isolates. Six conserved amino acid changes from the 2015 isolates were found in the mature viral proteins (VP3, 2B, 2C, 3A and 3D) in all 2020 isolates, excluding NADC1 (Table 3). The 12 protein coding regions in the six SVA isolates had completely identical protein sequences for VP4, VP2, 2A and 3B.

**Table 3:** Non-synonymous amino acid changes detected in 2020 SVA isolates as compared to 2015 U.S. isolates.

**Protein**	**Amino acid substitution**
VP3	V59E; A59E	
2B	G53A	I57V
2C	H290Q	
3A	S80T; G80T	
3D	D48G	

*Data excludes 2020 SVA genome NADC1*.

### Phylogenetic Analysis

**Figure 2:**
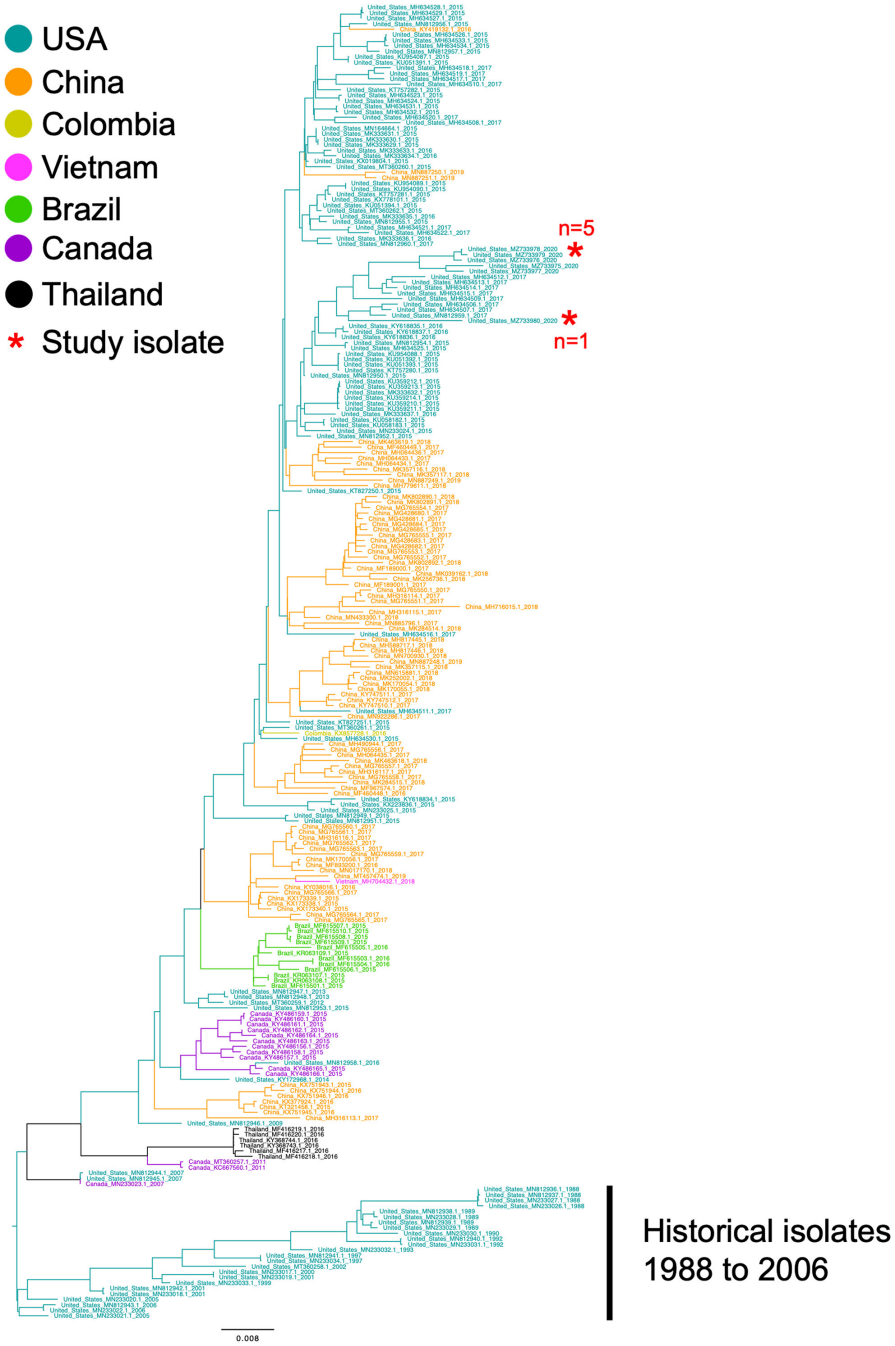
Phylogenetic analysis of 233 SVA whole genomes and the six 2020 SVA isolates from Plants 1-3. The tips of the tree are color coded to the country of sequence origin. The six SVA genome sequences analyzed in this study are noted with a red asterisk.

**Figure 3:**
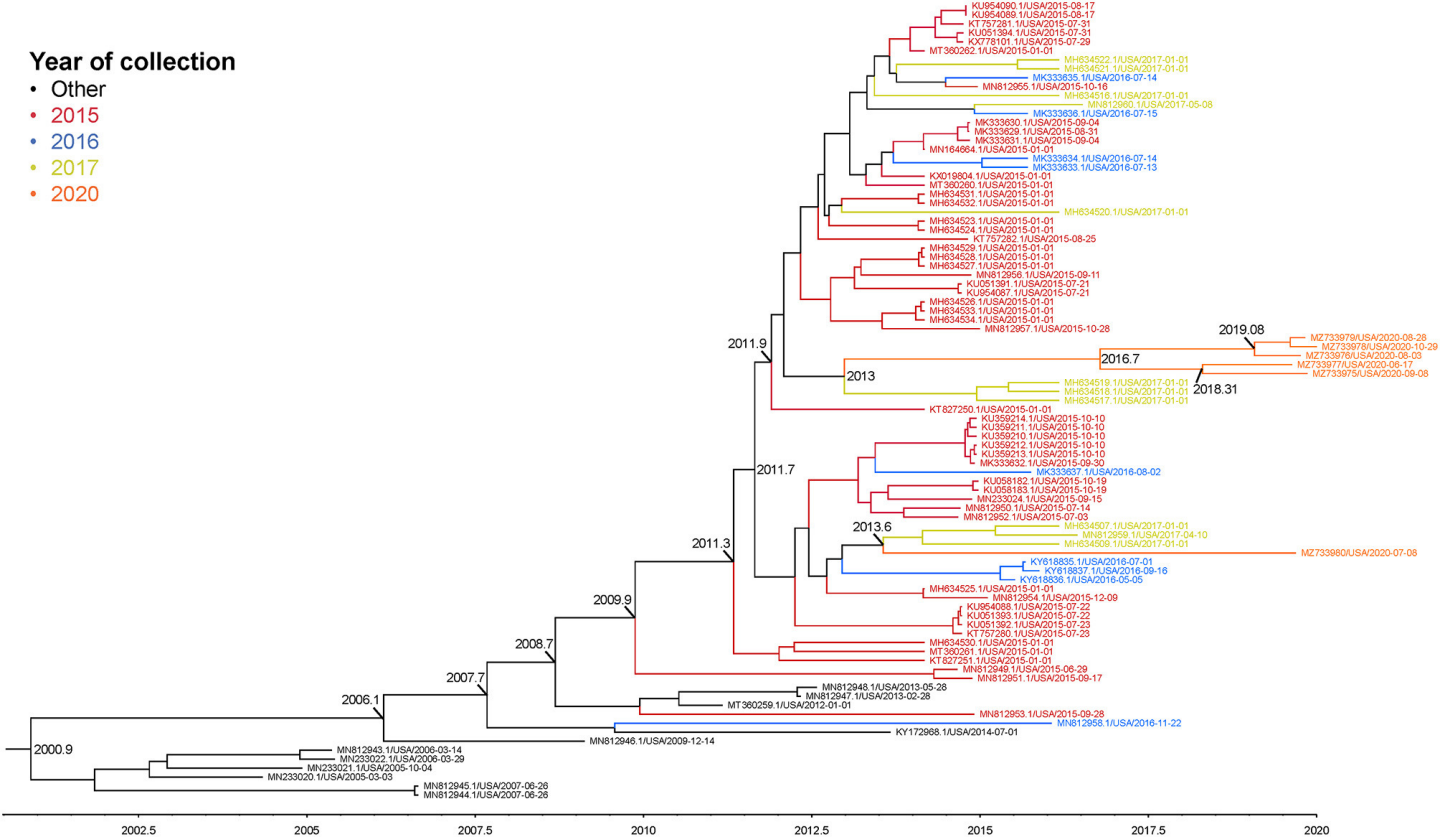
Maximum clade credibility (MCC) phylogenetic tree representing the evolutionary history of 83 SVA genome sequences and the six 2020 isolates. Colors indicate year of sample collection date. The x-axis indicates a timescale in years. Nodes are labeled with years of an estimated common ancestor.

The complete genome sequences of the six isolates in this study and 233 SVA whole genomes available from the U.S. Swine Pathogen Database (36) were analyzed. The analysis revealed that the historical SVA isolates from 1988 to 2006 formed a single monophyletic clade, and the contemporary SVA isolates were in a separate monophyletic clade with tree topology reflecting geographic origin and year of collection (Figure 2). Of the six SVA sequences collected in this study, five were located in a single clade with other 2020 strains: the sixth strain (NADC1) shared an evolutionary history with U.S. SVA strains isolated in 2017 (Figure 3). The most recent common ancestor (MRCA) for the six strains collected in this study was 2011.9 (2011.82–2012.3 95% HPD), and the MRCA for the five similar 2020 isolates was 2016.7 (2013.47–2021.06 95% HPD).

## Discussion

Vesicular lesions caused by SVA cannot be differentiated from those of FMD. As SVA continues to be endemic in the U.S, the number of FADIs related to swine vesicular lesions has been increasing (37). We investigated the presence of SVA in the environment of select U.S. sow slaughter plants, and quantified genetic diversity in the SVA genome in 2020 isolates relative to isolates collected in 2015.

SVA was found on multiple surfaces within slaughter plants with the flooring containing the highest percentage of SVA positive samples. Experimentally SVA has been demonstrated to be shed in feces multiple weeks after infection; therefore, it is likely that infected sows are shedding SVA in fecal material after arriving in holding pens at slaughter plants (14, 15). This could also explain why samples collected higher at the pig level like the gating and trailer swabs were less likely to be RT-qPCR positive for SVA. About a third of the SVA RT-qPCR positive environmental samples contained infectious SVA as determined by virus isolation. The lower number of VI positive samples compared to PCR positive samples may be the result of low levels of virus or inactivated virus due to time virus has been in the environment exposed to the elements, or the application of disinfectants in lairage. Numerically, a higher percentage of SVA RT-qPCR positive samples were found during warmer months. In contrast, other swine pathogens especially those involved with respiratory disease such as porcine respiratory and reproductive syndrome virus are diagnosed in a higher percentage during colder months in the fall and winter (38). Previous work has reported that house flies (*Musca domestica*) tested positive for SVA nucleic acids (17, 39); therefore, they could serve as a mechanical vector to spread SVA, which has been shown for other viruses and bacteria (40–42).

Previous studies have isolated SVA from environmental samples; however, there is limited information assessing the correlation between RT-qPCR results and virus isolation from environmental samples with infectivity of a pig (17, 18). Although samples with C_t_ values >30 were more likely to be VI negative there were several samples with high C_t_ values that were also VI positive. Since these samples were collected from lairage, they often contained fecal material; therefore, it is possible PCR inhibitors were present that may have impacted PCR C_t_ values (43). In this study, only one sample tested (C_t_ = 24.18) was positive by swine bioassay and capable of infecting piglets. A recent publication demonstrated that neonates are readily susceptible to SVA and had a minimum infectious dose of approximately 630 TCID_50_ for a 2011 SVA isolate (44). Due to the number of VI positive samples that did not infect piglets, the data in this study suggests that cell culture is more sensitive than swine bioassay. One evident explanation for VI being more sensitive than bioassay is the absence of innate immune barriers including mucus membranes, enzymes, stomach acid, and phagocytic cells. A better understanding of the infectivity of SVA found in the environment can help inform measures to reduce transmission and spread of SVA. In addition, previous work has shown the efficacy of some disinfectants including bleach (sodium hypochlorite) and accelerated hydrogen peroxide for inactivating SVA on various surfaces (45, 46).

Phylogenic analysis from this work showed one isolate (NADC1) did not group with the other five sequenced environmental samples. Our time-scaled phylogenetic analysis estimated the NADC1 sequence shared a most recent common ancestor with the other 2020 isolates in late 2011. The abundance of SVA positive samples from swine farms found throughout the United States combined with a range of reported sequences from 2015 until now suggest that a diversity of SVA may be cocirculating in U.S. swine herds (19, 47). Comparisons of the five most similar 2020 SVA isolates with the U.S. outbreak 2015 SVA isolates found six non-synonymous amino acid changes. Only the single amino acid change in the VP3 surface loop, “the knob,” is unique to the 2020 isolates (48). The other five amino acid changes were also identified in several 2017 U.S. SVA isolates suggesting these changes emerged prior to 2017. Experimentally infected pigs were shown to produce SVA specific IgG and IgM responses directed primarily against VP2 and VP3 (49). Although these amino acid changes could alter structural proteins, serum from experimentally inoculated pigs has been shown to have cross-neutralizing activity against geographically and temporally distinct SVA isolates (25).

While the majority of market-weight hogs are transported directly to slaughter plants, the sow market may include shipment to one or more collection points prior to reaching the slaughter plant (50). These points of congregation potentially housing sows from multiple sites likely act as areas of pathogen transmission and could explain the greater amounts of SVA found in the sow slaughter plants compared to the market-weight slaughter plant in this study (50). Since 2016, a majority of the vesicular disease FADIs were related to swine, with a large proportion of these due to the continued circulation of SVA in the U.S. (37). The presence of an endemic vesicular disease of swine is of great concern for growing complacency in FMD free countries (17–19). As such, it remains essential that producers and the market chain remain vigilant to avoid dismissing a case of FMD.

These data demonstrate that SVA can be detected in the environment of sow slaughter plants and these environmental isolates have the potential to infect naïve pigs. Our data on contemporary 2020 isolates demonstrated limited genetic diversity change relative to isolates from 2015; however, our surveillance of three sow slaughter plants detected two co-circulating genetic clades that diverged over ten years ago, and the observed genetic diversity between these strains suggests that phenotypic assessment and efficacy of vaccine control measures may need to be assessed relative to strains collected within the region. Future studies looking at SVA presence throughout the cull sow market chain and to sow farms of origin could provide valuable information about the continued circulation and transmission of SVA and guide measures to reduce the spread of SVA in the swine industry.

## Data Availability Statement

The datasets presented in this study can be found in online repositories. The names of the repository/repositories and accession number(s) can be found below: https://www.ncbi.nlm.nih.gov/genbank/, MZ733975-MZ733980.

## Ethics Statement

The animal study was reviewed and approved by NADC Animal Care and Use Committee in accordance with the Animal Care and Use Protocol (ACUP ARS-2018-750).

## Author Contributions

NH, JK, KL, and AB were responsible for the conception and design of the study. NH and AB were responsible for acquisition of the data. AB was responsible for animal and laboratory experiments. KH, TA, KF, and AB were responsible for data analysis and interpretation. KH was responsible for drafting the manuscript. AB and TA supervised manuscript editing. All authors read, revised, and approved the final manuscript.

## Funding

This work was supported in part by: the U.S. Department of Agriculture (USDA) Agricultural Research Service [ARS project number 5030-32000-230-000-D]; the U.S. Department of Agriculture (USDA) Animal and Plant Health Inspection Service, Veterinary Services [ARS project number 5030-32000-118-072-I]; and the USDA Agricultural Research Service Research Participation Program of the Oak Ridge Institute for Science and Education (ORISE) through an interagency agreement between the U.S. Department of Energy (DOE) and USDA Agricultural Research Service [contract number DE-AC05- 06OR23100].

## Conflict of Interest

The authors declare that the research was conducted in the absence of any commercial or financial relationships that could be construed as a potential conflict of interest.

## Publisher's Note

All claims expressed in this article are solely those of the authors and do not necessarily represent those of their affiliated organizations, or those of the publisher, the editors and the reviewers. Any product that may be evaluated in this article, or claim that may be made by its manufacturer, is not guaranteed or endorsed by the publisher.
